# Teleosts Genomics: Progress and Prospects in Disease Prevention and Control

**DOI:** 10.3390/ijms19041083

**Published:** 2018-04-04

**Authors:** Hetron Mweemba Munang’andu, Jorge Galindo-Villegas, Lior David

**Affiliations:** 1 Norwegian University of Life Sciences, Faculty of Veterinary Medicine and Biosciences, Department of Basic Sciences and Aquatic Medicine, Section of Aquatic Medicine and Nutrition, Adamstuen Campus, Ullevålsveien 72, P.O. Box 8146, Dep NO-0033, 0464 Oslo, Norway; 2 Department of Cell Biology and Histology, Faculty of Biology, Institute of Biomedical Research of Murcia-Arrixaca, Campus Universitario de Espinardo, University of Murcia, 30100 Murcia, Spain; jorge-galindo@um.es; 3 Department of Animal Sciences, R.H. Smith Faculty of Agriculture, Food and Environment The Hebrew University of Jerusalem P.O. Box 12, Rehovot 76100, Israel; lior.david@mail.huji.ac.il

**Keywords:** antimicrobial, disease, genomics, immunity, metagenomics, protection, vaccines

## Abstract

Genome wide studies based on conventional molecular tools and upcoming omics technologies are beginning to gain functional applications in the control and prevention of diseases in teleosts fish. Herein, we provide insights into current progress and prospects in the use genomics studies for the control and prevention of fish diseases. Metagenomics has emerged to be an important tool used to identify emerging infectious diseases for the timely design of rational disease control strategies, determining microbial compositions in different aquatic environments used for fish farming and the use of host microbiota to monitor the health status of fish. Expounding the use of antimicrobial peptides (AMPs) as therapeutic agents against different pathogens as well as elucidating their role in tissue regeneration is another vital aspect of genomics studies that had taken precedent in recent years. In vaccine development, prospects made include the identification of highly immunogenic proteins for use in recombinant vaccine designs as well as identifying gene signatures that correlate with protective immunity for use as benchmarks in optimizing vaccine efficacy. Progress in quantitative trait loci (QTL) mapping is beginning to yield considerable success in identifying resistant traits against some of the highly infectious diseases that have previously ravaged the aquaculture industry. Altogether, the synopsis put forth shows that genomics studies are beginning to yield positive contribution in the prevention and control of fish diseases in aquaculture.

## 1. Introduction

Teleosts fish species are one of the largest sources of protein for humans through their extensive production in aquaculture. There has been a growing trend in the last decades into the use of genomics to improve our disease control and prevention strategies against various diseases infecting different fish species in aquaculture. As such, genomics studies in aquaculture have evolved from reductionist single gene sequencing to high throughput next (HTS) generation sequencing, which has revolutionized our ability to read genomic data from entire metagenomes and transcriptomes [1,2,3]. These advances have transformed genomic studies into functional applications used for the discovery of genomic traits linked to disease resistance, discovery of novel pathogens, identification of novel probiotics and antimicrobial compounds [4,5,6,7,8,9,10,11]. In vaccinology, genomics studies are being used in search of immune genes that correlate with protective immunity to serve as benchmarks in optimizing vaccine efficacy [12,13,14,15]. In therapeutics they are used to identify antimicrobial peptides (AMPs) needed to prevent pathogens invasion and remedial genes needed for tissue regeneration [16,17,18,19]. Altogether, these advances show that genomics studies are being transformed into functional applications contributing to reducing the disease burden in fish farming. In the current review, we provide an overview of the progress and prospects of functional genomics based on conventional single gene sequencing, cloning and characterization together with HTS in the prevention and control of fish diseases in aquaculture [20].

## 2. Metagenomics Analyses

Traditional diagnostic methods used for the identification of infectious agents depend on isolation, culture, characterization, immunoassays and PCR [21]. When these methods fail, enhanced molecular techniques such as nested, multiplex, differentially labelled probes or consensus PCRs using degenerate primers are used [22,23,24,25,26]. In some cases, enhanced molecular techniques also fail to identify novel pathogens resulting in emerging diseases to reach epidemic proportions before the etiological is identified. However, the upcoming of metagenomics, which is a proactive diagnostic approach that does not require prior knowledge of the infectious agent to be diagnosed has accelerated our ability to identify novel pathogens. Currently, there are two approaches used for metagenomics analysis namely (i) deep amplicon sequencing (DAS) that uses pre-sequencing PCR to amplify a selected taxonomic marker such as the 16S rRNA, and (ii) shotgun pyrosequencing in which all nucleic acids present in the sample are sequenced and de novo assembled [21]. Both approaches have been widely used in the aquaculture. 

### 2.1. Shotgun Pyrosequencing

Shotgun pyrosequencing has contributed to the discovery of more than 40 viral diseases in different aquatic organism in the last decade, which is by far more than the number of viral diseases discovered using the traditional methods in the last 50 years [27]. The duration at which novel viruses are being discovered using this approach is shorter than the period taken by traditional diagnostic methods in which etiological agents are often discovered after the disease has reached epidemic proportions [28]. By enhancing our ability to identify novel pathogens, metagenomics expedites our ability to develop timely disease control strategies. In terms of diagnosis, it has the advantage of being able to identify etiological agents of single and co-infections involving several etiological agents infecting the same fish enabling the selection of the appropriate treatment [29,30,31]. It is also used to identify epidemiological factors linked to the spread of different pathogens through ballast water, global aquaculture trade, sale of fish and fish-by-products and migratory aquatic organisms across the world there by facilitating the design of effective transboundary disease control strategies and biosafety control measures [27,32,33,34,35]. It has also been used to evaluate the composition of pathogens that resist disinfection in closed recirculation systems indicating that it can be used for optimizing disinfection procedures for recirculation systems [36]. As the cost of shotgun pyrosequencing continues to decrease, we anticipate that metagenomics will become a part of routine diagnosis and epidemiological surveys in aquaculture.

### 2.2. Deep Amplicon Sequencing

Deep amplicon sequencing (DAS) using a guided taxonomic marker such as 16S rRNA has been used to study the microbial composition of different aquatic environments as an overture to designing to effective preventive measures against endemic fish diseases in each ecosystem. The bacterial composition on the skin surface has been shown to play a major role in protecting fish against pathogen invasion. Boutin et al. [37] used the metagenomics analysis to show changes on the skin surface microbiome. They noted that a shift from Methylobacteria dominated population, which is known to protect the skin surface, to pathogenic species such as Flavobacterium was indicative of susceptibility to infection clearly demonstrating the skin health status can be monitored based on changes in the surface microbial composition. Similarly, Gajardo et al. [38] used metagenomics to identify bacterial species that induce diet dysfunction to serve as biological markers for monitoring the gut health status of Atlantic salmon (*Salmo salar* L.). 16S rRNA metagenomics can be used to identify probiotics for use in fish as well being as a way of monitoring the gut microbiota changes induced by different feed formulations [39]. The ratio of Firmicutes to Bacteriodetes has been used as a measure of the health index in vertebrates [40,41] implying metagenomics analyses can be used to monitor the health indices of culture fish species based on the composition of gut microbiota. Metagenomics analysis can be used to monitor the safety of aquatic environments used in aquaculture by providing an overview of the prevailing microbial composition, predict the susceptibility of the skin to microbial invasion, determine the health status of the gut and provide the health index of fish based on the gut microbiota composition.

## 3. Antimicrobial Peptides (AMPs) and Antiviral Compounds

Masso-Silva and Diamon [42] have shown that fish are a great source of antimicrobial peptides (AMPs) that include defensins, cathelicidins, hepcidins, histone derived peptides and piscidins. In general, fish AMPs have broad spectrum antimicrobial properties able to kill both fish and mammalian pathogens and they also have immunodulatory properties [42]. Masso-Silva and Diamon [42] have provide a comprehensive list of fish species in which these AMPs have been detected [42]. However, in the current review, we highlight the use of fish AMPs in disease control and prevention using examples carried out in different studies. Although most AMPs discovered this far have not been commercialized, indications are that they have the potential to play an important role in disease control and prevention in aquaculture.

### 3.1. Hepcidins

Huang et al. [43] used phage hybridization to identify three hepcidin-like antimicrobial peptides from *Oreochromis mossambicus* (tilapia) namely Th1-5, TH2-2, and TH2-3. These peptides have antimicrobial properties against bacteria, fungi and viruses [43] and they have been identified, cloned and characterized in different fish species [44,45,46]. They have immunomodulatory functions in fish and they have been shown to produce antitumor effects in human cells [16]. Wang et al. [18] showed high protection against nervous necrosis virus (NNV) using hepcidin1-5 in medaka (*Oryzias latipes*). They also showed that the level of protection was dose dependent in which fish injected with 1.0 μg, 0.5 μg, 0.01 μg, 0.01 μg/fish had protection levels of RPS = 86, 68, 59 and 48%, respectively.

As for application against bacterial infections, hepcidins produce antimicrobial effects against a wide range of extracellular infections. For example, Liu et al. [44] characterized the functional analysis of the hepcidin gene in roughskin sculpin (*Trachidermus fasciatus*) and showed that it exhibited a wide spectrum of potent antimicrobial activity against *Escherichia coli*, *Vibrio Anguillarum*, *Klebsiella pneumoniae*, *Pseudomonas aeruginosa*, *Staphylococcus aureus*, *Bacillus subtilis*, *B. thuringiensis*, and *B. megaterium* with minimum inhibitory concentrations (MICs) of 5–80 μg/mL (0.825–13.2 μM). In taimen (*Hucho taimen*, Pallas) it showed antimicrobial activities against *Micrococcus lysodeikticus* and *S. aureus* and *E. coli* [45] while in spotted scat it showed antiviral activities against *Siniperca chuatsi* rhabdovirus (SCRV) and largemouth bass Micropterus salmoides reovirus (MsReV) as well as a wide range of Gram-negative and positive bacteria species [47].

Apart from showing antimicrobial properties against extracellular bacteria, hepcidin also shows antimicrobial properties against intracellular bacteria as shown by Chen et al. [48] that Hepcidins 1 and 2 had the capacity to hydrolyze purified genomic bacteria DNA for *Edwardsiella tarda* infection in Mudskipper (*Boleophthalmus pectinirostris*). Similarly, Alvarez et al. [49] showed that the oxidized hepcidin peptide was more effective than the reduced peptide against *Piscirikettsia salmonis*, which is an intracellular bacterium shown to cause high mortalities in salmonids. Therefore, these observations show that AMPs could serve as therapeutic agents against intracellular bacterial infections that have posed a significant challenge in vaccine development in aquaculture.

### 3.2. Epinecidin-1

Epinecidin-1 is an important AMP originally isolated from a complementary cDNA library of grouper (*Epinephelus coioides*) having a broad range of antimicrobial properties against bacteria, fungi and viruses [17,50]. It has also been characterized from other fish species such as *Epinephelus akaara* [51]. In human cells, it induces apoptosis that enhances antitumor effects as well as immunomodulatory effects by increasing the expression of different cytokines such as TNFα, IL-10, IL-6 and IFN [52]. Wang et al. [53] examined the efficacy of pre-, co- and post-treatment effects of epinecidin-1 against NNV and showed that co-treatment of epinecidin-1 with NNV decreased grouper mortality. They also observed that challenge with NNV in co-treated fish increased fish survival. In addition, they also showed that fish inoculated with NNV followed with epinecidin-1 treatment showed significantly higher protection suggesting that epinecidin-1 can be used as a drug to rescue infected grouper. Immunohistochemistry staining showed that epi-1 treatment during or after infection cleared off the virus. Apart from increasing survival in fish exposed to viral infections, it has also been shown to increase the survival of fish exposed to bacterial infections such as *Vibrio vulnificus* in grouper and zebrafish [54].

### 3.3. Other Antimicrobial Peptides

Other AMPs identified include piscidins, β-defensins and Cathelicidins [42,55,56,57]. Piscidins have antimicrobial activities against a wide range of Gram-positive bacteria inclusive of Streptococci species and Gram-negative bacteria species such as the *Vibrio* species. In addition, they possess antiviral properties against viruses such as channel catfish virus [58], anti-fungal properties against *Psuedosciaena crocea* [59] and *Candida albican* [60] and anti-parasitic properties against *Cryptocaryon irritans* and *Ichthyophthirius multifiliis* [59,61]. Similarly, β-defensins have antibacterial properties against *A. hydrophila* [62], and antiviral properties against a wide range of viruses such as viral hemorrhagic septicemia virus (VHSV), Singapore grouper iridovirus (SGIV) and NNV [63,64,65]. The cathelicidins have been shown to produce antibacterial properties against *Yersinia ruckeri* [66] and antifungal properties against *Candida albicans* [67].

### 3.4. Interferon

Interferons (IFNs) are antiviral compounds known to prevent virus infection whose protective role has been demonstrated in different fish species. Ooi et al. [68] have shown that IFNα-2 is protective against infectious hematopoietic necrosis virus (IHNV) in rainbow trout (*Oncorhynchus mykiss*) and that protection was dose-dependent with highest relative percent survival (RPS = 90%) being from a dosage of 1.0 g/fish while the 0.1 μg/g dose had a low RPS (39%). Kuo et al. [69] showed that increasing the oral dosage of IFN encapsulated in PLGA nanoparticles from 0.05 to 0.1 μg of PLGA-IFNγ/fish, increased the RPS from 12.4 to 46.7%. Similar results have been reported in stickleback (*Gasterosteus aculeatus*), medaka and European sea bass (*Dicentrarchus labrax*) treated with recombinant IFN [70]. However, as pointed out by Ooi et al. [68] the duration of protection induced by IFN treatment is shorter than that produced by humoral responses and, hence, this would account for reasons why IFNs are not widely used as therapeutic agents in aquaculture. Moreover, not all pathogens succumb to IFN treatment as shown by Gadan et al. [71] that infectious pancreatic necrosis virus (IPNV) was able to replicate at a higher rate in the presence of type I IFN in Atlantic salmon (*Salmo salar* L.) subjected to stress conditions. At cellular level, IFN produces immunomodulation effect as shown by Ooi et al. [68] that IFNa-2 treatment in rainbow trout increased systemic expression of several IFN-induced genes (ISGs) such as IFN1, IRF 1 and 2, MHC-I, STAT1 and vig-1. Similarly, Xu et al. [3,72,73] used transcriptome analyses to show that type I IFNs modulates the expression of several antiviral genes and activate several signaling pathways that lead to the production of several other antiviral genes.

### 3.5. Therapeutic Compounds

Apart from the discovery of AMPs targeted at protecting fish from invading pathogens, current advances in genomic studies are paving the way into the discovery of remedial peptides useful for tissue regeneration. For example, Hoppe et al. [19] used transcriptome analysis to identify the differentially expressed genes (DEGs) after renal damage caused by gentamicin in killifish (*Nothobranchius furzeri*). Among the DEGs was miR-21, which was upregulated and was shown to be essential for kidney regeneration in killifish. In fish treated with gentamicin, miR-21 upregulation was blocked by injecting anti-miR-21 prior to induction of renal injury. They noted that apoptosis in renal tubules was caused by miR-21 inhibition while its upregulation positively influenced the initiation of renal tubule regeneration. Hence, it is likely that as studies on transcriptome analyses in search of remedial peptides continues, more genes will be discovered linked to tissue regeneration in fish.

## 4. Application of Genomic Studies in Vaccine Development

In vaccine development, genomics studies have dual functions of identifying protective antigens and elucidating the underlying mechanisms of immune protection induced by vaccination. Several approaches have been used for the search of immunogenic proteins for viral and bacterial vaccines. In the case of viral vaccines, reverse genetics have been used for the identification of immunogenic proteins for IPNV [74], peptide scanning for NNV [75] and IPNV [76], phage display for infectious salmon anemia virus (ISAV) [77] and IHNV [78] while bioinformatics has been used to identify immunogenic proteins for SGIV [79]. The search of bacterial immunogenic proteins is mostly centered on identifying antigens that provide protective immunity across variant serotypes of the same bacterial species. Wang et al. [80] used the tandem mass tag (TMT) labelling-based quantitative proteomics to identify the most immunogenic outer membrane proteins (OMPs) of *A. hydrophila*. Another approach used for bacterial vaccines is the in vivo induced antigen technology (IVIAT), which has been widely used for the selection of antigens for different bacterial species such as *Edwardsiella tarda* [81], *A. salmonicida* [82] and *Streptococcus iniae* [83]. Taken together, these advances play an important role in the design of genetically engineered vaccines encoding immunogenic proteins in heterologous vectors used in the production of DNA, fusion protein and subunit recombinant vaccines. Apart from genetically engineered vaccines, genomic studies have also been used for the identification of avirulent strains for use as live attenuated vaccines as shown by Zhang et al. [84] who used Illumina sequencing for the characterization of attenuated strains of *Streptococcus agalactiae* with the potential to serve as a live vaccine.

In fish vaccinology, transcriptome studies are being used to elucidate the mechanisms underlying vaccine protection. For example, Zhang et al. [85] used transcriptome analyses to show that early immune responses in Large yellow croaker (*Larimichthys crocea*) vaccinated against *Vibrio parahaemolyticus* and *A. hydrophila* were dominated by innate defense molecules involved in antigen uptake and inflammasome formation. RNA-seq data generated by Yan et al. [86] showed an activated MHC-I and inhibited MCH-II pathway in zebrafish immunized with a live attenuated *E. tarda* vaccine during the early stages of immune response to vaccination. Yasuike et al. [87] identified five interferon-inducible genes (ISG) inclusive of Mx that correlated with high protection induced by the G-protein encoded in the DNA vaccine used to immunize Japanese flounder against Hirame rhabdovirus (HIRRV). Similarly, Liu et al. [88] used deep sequencing to identify a repertoire of seven genes comprising of IFN-I, TNFα, CRP, IL-8, IgM, MHC-I and CD8α that correlated with high protection in grass carp (*Ctenopharyngodon idella*) vaccinated against *A. hydrophila* using a live recombinant vaccine while Bridle et al. [15] identified a biosignature dominated by IgH and selenoprotein predictive of the level of protection in Atlantic salmon vaccinated against *Yersinia ruckeri* by immersion. Other genes that have been shown to correlate with vaccine protection include GATA-3 expressed in Atlantic salmon vaccinated against IPNV [12] and Mx in rainbow trout vaccinated against viral hemorrhagic septicemia virus (VHSV) [89]. Even though antibodies are the most widely used measure of immune response to vaccination in fish [13,90,91,92] they cannot be used as correlates of protection for cytotoxic T-lymphocyte (CTL) responses [93]. Hence, the use of transcriptome analyses does not only provide a global understanding of the host immune response to vaccination, but it unravels gene markers that correlate with cellular-mediated immune responses for use as benchmarks in vaccine development.

## 5. Application of Functional Genomics in Disease Control

### 5.1. Genetic Markers of Disease Resistance and Susceptibility

Different approaches have been used for the identification of disease resistance and susceptibility traits in fish. These include the random fragment length polymorphisms (RFLP), random amplified polymorphism DNA (RAPD), amplified fragment length polymorphism (AFLP) and quantitative trait loci (QTL) [94]. In Norway, identification of a QTL for resistance against IPNV in Atlantic salmon has led to the significant decrease in the number of IPNV cases reported annually from more than 200 cases in 2005 to less than 30 cases in 2016 [95] clearly demonstrating that QTL analysis can reliably be used to identify disease resistance traits for diseases having a devastating economic impact in aquaculture. Table 1 shows this approach is being explored for the search of markers against other several diseases in aquaculture.

Apart from disease resistance traits, genetic markers for susceptibility have also made positive contributions to disease control in aquaculture. Fish that are highly susceptible to disease infection have found a positive contribution in vaccine production as one of the most important elements required for optimizing challenge models needed in vaccine efficacy trials. As pointed out previously [13,14,115], for a challenge model to be reproducible and reliable, it must have a wide discriminatory capacity between the vaccinated and control fish. Given that relative percent survival (RPS) is the most widely used measure of vaccine efficacy, fish used in challenge models must show high mortality after lethal challenge. Hence, genetic selection for susceptibility traits serves as an important tool for identifying fish strains required for developing optimal challenge models needed for vaccine efficacy trials. Recently, we used a highly susceptible strain of Atlantic salmon in optimization the cohabitation challenge model for IPNV in which we obtained high mortality in unvaccinated fish (>80%) in three independent studies leading to a wide discriminatory capacity (RPS > 50%) between the vaccinated and control fish [115]. These studies demonstrate that genomic studies are being used in vaccine production and disease control strategies in aquaculture while fish that are highly susceptible to disease infection are used in optimization of vaccine performance. 

### 5.2. Immunological Markers of Disease Resistance

Different immune parameters such as the major histocompatibility (MHC) molecules can be used for the selection of disease susceptibility and resistance traits in fish. MHC variants influence several vital biological traits such as susceptibility to infection, autoimmunity and immune recognition governed by their ability to encode cell surface glycoproteins that bind peptides processed for presentation to T-lymphocytes [116]. Different studies have shown that the loci that code for peptide binding on MHC-molecules are among the most polymorphic loci in vertebrates [116]. As shown in Table 2, the association between MHC polymorphism and disease resistance has been reported in different fish species. Xu et al. [117] found a single allele linked to resistant to *Vibrio anguillarum* infection in 12 selective Japanese flounder families. In another study, Yang et al. [118] identified a single nucleotide polymorphism (SNP) associated with resistance against Singapore grouper iridovirus (SGIV) in Orange spotted grouper (*Epinephelus coioides*). To confirm this, a fish encoding the highly resistant (HR) and less resistant (LR) SNP were infected with SGIV resulting in low mortality in the HR compared to LR fish. Similarly, Palti et al. [119] found an association between SNPs tightly linked to MHC-II and IHNV resistance in backcrosses of rainbow trout and cutthroat trout (*Oncorhynchus clarkii*).

Apart from MHC molecules, SNPs have been reported in other immune genes such as the toll like receptors (TLRs) and retinoic acid inducible gene I (RIG-) like receptors (RLRs), which are germline coded pathogen recognition receptors (PRRs) involved in the recognition of pattern associated molecular patterns (PAMPs) expressed by different pathogens. As shown in Table 2, SNPs on PRRs have been associated with disease resistance and susceptibility traits. For example, Heng et al. [128] selected eight SNPs in the non-coding region of TLR3 and analyzed the genotype allele distribution of susceptibility and resistance traits of carp to grass carp reovirus (GCRV) infection using PCR-RFLP. They found one allele, which was significantly associated with resistance to GCRV. To confirm the correlation, they carried out a challenge study, which showed significantly low mortality in fish encoding the disease resistance SNP. Similarly, Wang et al. [130] identified six SNPs on the MDA5 genome of grass carp using PCR-RFLP and examined their association with resistance to GCRV. They found two loci linked to resistance against GCRV and carried out verification challenge studies in which the HR group had significantly low mortality unlike the LR group that had high mortality. Other PRRs shown to have SNPs linked to disease resistance against GCRV are TLR22, RIG-I like and LGP2 [131,132,136]. Apart from PRRs, Kongchum et al. [126] found an association between IL-10a SNP and resistance to Cyprinid herpesvirus infection in common carp (*Cyprinus carpio*).

## 6. Conclusions

In general, genomics studies in aquaculture have progressively been transformed into functional applications aimed at improving the quality of fish products by contributing to reducing the disease burden in aquaculture. Metagenomics analyses focus on identifying novel pathogens, epidemiological monitoring of the microbiome composition of aquatic environments used for aquaculture and establishing biological markers for monitoring skin and gut health status based on microbiota composition. Discovery of a wide range of AMPs as therapeutic drugs has been attained for different fish species with prospective applications that would contribute to reduction of antibiotic use in aquaculture. Progress in vaccine development has not only led to discovery of various immunogenic proteins essential for optimizing vaccine performance, but is slowly being turned into vaccine efficacy optimization using genomics by trying to identify gene signatures that correlate with protective immunity for use as measures of efficacy in vaccine production. Finally, single nucleotide polymorphisms and QTL mapping are showing great prospects in disease prevention and could lead to disease eradication in some parts of the world as shown in the use of QTL resistance traits for IPNV in Atlantic salmon, which has significantly reduced the number of cases of this devastating disease in Norway. In summary, these observations show that current advances in genomics studies are positively contributing to prevention and control of fish diseases in aquaculture.

## Figures and Tables

**Table 1:** Traits for disease resistance determine by quantitative trail loci mapping.

Disease/Pathogen	Fish Species	Reference
Infectious salmon anemia	Atlantic salmon (*Salmo salar* L.)	[8]
*Flavobacterium psychrophilum*	Rainbow trout (*Oncorhynchus mykiss*)	[9]
Viral hemorrhagic scepticemia virus	Rainbow trout (*Oncorhynchus mykiss*)	[10]
Grass carp reovirus	Grass carp (*Ctenopharyngodon idella*)	[96]
Columnaris	Channel catfish (*Ictalurus punctatus*)	[97]
*Vibrio anguillarum*	Japanese flounder (*Paralichthys olivaceus*)	[98]
*Aeromonas hydrophila*	Rohu (*Labeo rohita*)	[99]
Coldwater disease	Rainbow trout (*Oncorhynchus mykiss*)	[100]
Viral haemorrhagic scepticemia	Turbot (*Scophthalmus maximus*)	[101]
Monogenean (*Benedenia seriolae*)	Yellowtail (*Seriola quinqueradiata*)	[102]
*Philasterides dicentrarchi*	Turbot (*Scophthalmus maximus*)	[103]
Whirling disease	Rainbow trout (*Oncorhynchus mykiss*)	[104]
Pasteurellosis	Gilhead sea bream (*Sparus aurata*)	[105]
Infectious pancreatic necrosis	Atlantic salmon (*Salmo salar* L.)	[7,106,107,108,109,110]
*Gyrodactylus salaris*	Atlantic salmon (*Salmo salar* L.)	[111]
Lymphocystis disease	Japanese flounder (*Paralichthys olivaceus*)	[112,113]
Salmonid alphavirus	Atlantic salmon (*Salmo salar* L.)	[114]

**Table 2:** Immune markers of disease resistance.

Gene	Disease/Pathogen	Fish Species	Reference
MHC-IIB	Cold water	Rainbow trout (*Oncorhynchus mykiss*)	[120]
MHC-IIB	*Vibrio anguillarum*	Japanese flounder (*Paralichthys olivaceus*)	[117]
MHC-IIB	*Edwardsiella tarda*	Turbot (*Scophthalmus maximus*)	[121]
MHC-IIB	*Vibrio anguillarum*	Half-smooth tongue sole (*Symphurus thermophilus)*	[122]
MHC-IIB	Singapore iridovirus	Orange spotted grouper (*Epinephelus coioides*)	[118]
MHC-II	Piscirickettsia	Atlantic salmon (*Salmo salar* L.)	[123]
MHC-II	*Aeromonas salmonicida*	Atlantic salmon (*Salmo salar* L.)	[124,125]
MHC-I and II	Infectious anemia virus	Atlantic salmon (*Salmo salar* L.)	[124]
MHC-II	Infectious hematopoietic necrosis	Rainbow trout (*Oncorhynchus mykiss*)	[119]
MHC-II	Infectious hematopoietic necrosis	cutthroat trout (*Oncorhynchus* *clarki*)	[119]
IL-10B	Cyrprinid herpesvirus CyHV-3	Common Carp (*Cyprinus carpio*)	[126]
TLRs	Cyrprinid herpesvirus (CyHV-3)	Common Carp (*Cyprinus carpio*)	[127]
TLR3	Grass carp reovirus	Grass carp (*Cyprinus carpio*)	[128]
TLR22	Grass carp reovirus	Grass carp (*Ctenopharyngodon idella*)	[129]
MDA5	Grass carp reovirus	Grass carp (*Ctenopharyngodon idella*)	[130]
RIG-I	Grass carp reovirus	Grass carp (*Ctenopharyngodon idella*)	[131]
LGP2	Grass carp reovirus	Grass carp (*Ctenopharyngodon idella*)	[132]
Lysozyme	*Vibrio harvey*	Asian seabass (*Lates calcarifer*)	[133]
Lysozyme	Photobacterium	Asian seabass (*Lates calcarifer*)	[133]
LECT2		Asian seabass (*Lates calcarifer*)	[134]
Ceruloplasmin	*Aeromonas hydrophila*	Rohu (*Labeo rohita*)	[135]
